# GraphKM: machine and deep learning for K_M_ prediction of wildtype and mutant enzymes

**DOI:** 10.1186/s12859-024-05746-1

**Published:** 2024-03-28

**Authors:** Xiao He, Ming Yan

**Affiliations:** College of Biotechnology and Pharmaceutical Engineering, Nanjing Tech University, Nanjing, China

**Keywords:** Neural networks, Tree boosting, Michaelis constant, Deep learning, Graph neural network

## Abstract

Michaelis constant (K_M_) is one of essential parameters for enzymes kinetics in the fields of protein engineering, enzyme engineering, and synthetic biology. As overwhelming experimental measurements of K_M_ are difficult and time-consuming, prediction of the K_M_ values from machine and deep learning models would increase the pace of the enzymes kinetics studies. Existing machine and deep learning models are limited to the specific enzymes, i.e., a minority of enzymes or wildtype enzymes. Here, we used a deep learning framework PaddlePaddle to implement a machine and deep learning approach (GraphKM) for K_M_ prediction of wildtype and mutant enzymes. GraphKM is composed by graph neural networks (GNN), fully connected layers and gradient boosting framework. We represented the substrates through molecular graph and the enzymes through a pretrained transformer-based language model to construct the model inputs. We compared the difference of the model results made by the different GNN (GIN, GAT, GCN, and GAT-GCN). The GAT-GCN-based model generally outperformed. To evaluate the prediction performance of the GraphKM and other reported K_M_ prediction models, we collected an independent K_M_ dataset (HXKm) from literatures.

**Supplementary Information:**

The online version contains supplementary material available at 10.1186/s12859-024-05746-1.

## Introduction

Machine and deep learning have been applied and shown application in the fields of biology, such as Golgi proteins classification [1], Lysine 2-hydroxyisobutyrylation identification [2], and kinetic models [3–6]. The Michaelis constant (${K}_{M}$) describes the affinity of an enzyme for a specific substrate [7]. And the ${K}_{M}$ is one of central parameters for enzyme kinetics in the fields of protein engineering, enzyme engineering, and synthetic biology. As overwhelming experimental measurements of ${K}_{M}$ are difficult and time-consuming, predictions of the ${K}_{M}$ value from artificial intelligence would increase the pace of enzyme kinetics research [7].

Existing machine and deep learning models are limited to the specific enzymes, i.e., a minority of enzymes or wildtype enzymes. In 2006, Borger et al. trained a linear model to predict ${K}_{M}$ values [8]. The dataset is composed of the ${K}_{M}$ measurements for the same substrate paired with different enzymes in the same organism and with the same enzymes in other organisms. They fitted the model for each of 8 different substrates [8]. In 2012, Yan et al. reported a ${K}_{M}$ prediction model of beta-glucosidases for the substrate cellobiose based on a deep learning method [9]. The dataset contains 36 matched sequences and ${K}_{M}$ values of β-glucosidases [9]. Until 2021, Kroll et al. used the deep learning framework Tensorflow to implement a ${K}_{M}$ prediction model (namely KM_prediction) for wildtype enzyme–substrate combinations [7]. The dataset used for this model has 11,675 entries with wildtype enzymes [7]. In learning model selection, they compared various methods, elastic net, gradient boosting model, and fully connected neural network (FCNN). The gradient boosting model performed better [7]. In substrate representation, they compared the substrate ECFP, MACCS keys, RDKit molecular fingerprints, and graph neural networks (GNN) fingerprints [7]. The GNN fingerprints performed better fitting gradient boosting model separately with ${K}_{M}$ values [7]. A global feature vector concatenated the vector learned with the GNN and the vector generated by UniRep50 (a tool generating protein sequence representation) was used as the input for the gradient boosting model to predict the ${K}_{M}$ value. The KM_prediction model achieved the training results of MSE = 0.653 and ${R}^{2}$ = 0.527 [7]. In 2022, Maeda et al. reported a ${K}_{M}$ prediction model for wildtype enzymes [10]. The dataset used for this model has 17,151 entries (one entry contains EC number, Kegg Compound ID, and Organism ID) [10]. In model selection, they compared various methods, k-nearest neighbors, elastic net, random forest, gradient boosting, and TabNet. The random forest model performed better [10]. The representation used for the model is a concentration of the one-hot encodings of EC number, Kegg Compound ID, and Organism ID. Their model achieved prediction scores: RMSE = 0.795 and R^2^ = 0.536 [10].

Neural networks that operate on graphs have been previously introduced by Gori et al. [11] and Scarselli et al. [12]. Many variants of GNN have been reported at present, such as graph isomorphism networks (GIN) [13], graph attention networks (GAT) [14], graph convolutional neural networks (GCN) [15], etc. The original GIN uses a multi-layer perceptron model to update the node features [16]. The original GAT proposes an attention-based architecture to learn hidden representations of nodes in a graph by applying a self-attention mechanism. The original GCN is designed for semi-supervised node classification problem, i.e., the model learns the node-level feature vectors [17].

The sequences of proteins at the scale of evolution contain an image of biological function. The biological properties of a protein constrain the mutations to its sequence that are selected through evolution, recording biology information into evolutionary patterns [18–20]. Protein function can therefore be inferred from the patterns in sequences [21]. As the representational capacity of the language model and the diversity of protein sequences seen in its training increase, deep information about the biological properties of the protein sequences will emerge [22]. ESM-2, in variants up to 15 billion parameters, is a transformer-based language model, and uses an attention mechanism to learn interaction patterns between pairs of amino acids in the input sequence [22].

Tree boosting is a highly effective and widely used machine learning method [23]. Gradient boosting of regression trees produces competitive, highly robust, interpretable procedures for both regression and classification, especially appropriate for mining less than clean data [24]. XGBoost, a scalable end-to-end tree boosting system, is used widely by data scientists to achieve state-of-the-art results on many machine learning challenges [23]. It implements machine learning algorithms under the gradient boosting framework.

There are a lot of frameworks for deep learning, such as TensorFlow, PyTorch, and PaddlePaddle. TensorFlow features Keras as a high-level API which abstracts away a lot of underlying code making it easier and faster to create and train models. PyTorch provides a strong and flexible API to work with CPU and GPUs. PyTorch’s excellent support for GPUs makes distributed training more optimized and feasible. Paddlepaddle maintains both high runtime performance and development flexibility.

In this study, we used a deep learning framework PaddlePaddle to implement a machine and deep learning approach, namely GraphKM, to predict wildtype and mutant enzyme–substrate Michaelis constants. In contrast to previous, we represented the substrates through molecular graph and the enzymes through a pretrained transformer-based language model to construct the model inputs. We compared the difference of the model results made by different GNN (GIN, GAT, GCN, and GAT-GCN). We showed the prediction performance of GraphKM on an independent dataset collected from literatures.

## Methods

### Data cleaning

The dataset was extracted from the BRENDA [25] and SABIO-RK [26] databases on 31 August 2023 by customized scripts invoking application programming interface.

The initial dataset only contained the substrate name, organism information, EC number, UniProt ID (incomplete), enzyme type and ${K}_{M}$ value. The substrate SMILES codes were extracted from querying the compound database PubChem [27] using substrate name and were saved in an independent json file. Protein sequences were queried in two ways: for entries with UniProt ID, the amino acid sequences were obtained via querying UniProtKB [28] website; and for entries without UniProt ID, the amino acid sequences were acquired from the BRENDA [25] and UniProtKB [28] databases based on their EC number and organism information. The queried sequences were saved in an independent json file.

We ensured that the same canonical SMILES codes were output for the same substrates with various synonyms. The sequences of wildtype enzymes were mapped in the initial dataset directly, and the sequences with mutated sites were changed according to the mutated information. As several entries with the same amino acid sequence and substrate have multiple ${K}_{M}$ values, we reserved the entry with the maximum ${K}_{M}$ value. Entries with missing information and redundant entries were filtered out. To ensure quality, several rounds of data cleaning were performed (Additional file 1: Fig. S1).

As the enzymes with more than 1,000 AA are fusion proteins, we removed the entries with enzymes (protein sequence length ≧1,000 AA). And we log10-transformed all ${K}_{M}$ values. The cleaned dataset is a comprehensive dataset including the substrate name, substrate SMILES, organism information, EC number, amino acid sequence, enzyme type and ${K}_{M}$ value.

### Data preprocessing

The substrate SMILES code was converted to its corresponding molecular graph by the open-source chemical informatics software RDKit [29] (converting the SMILE code into the mol information) and the PaddleHelix package (https://github.com/PaddlePaddle/PaddleHelix) (converting the mol information into the molecular graph).

The protein sequence was converted to a 1,280-dimensional vector by the pretrained ESM-2 (650 M) model (https://github.com/facebookresearch/esm).

### The framework of the GraphKM model

**Fig. 1:**
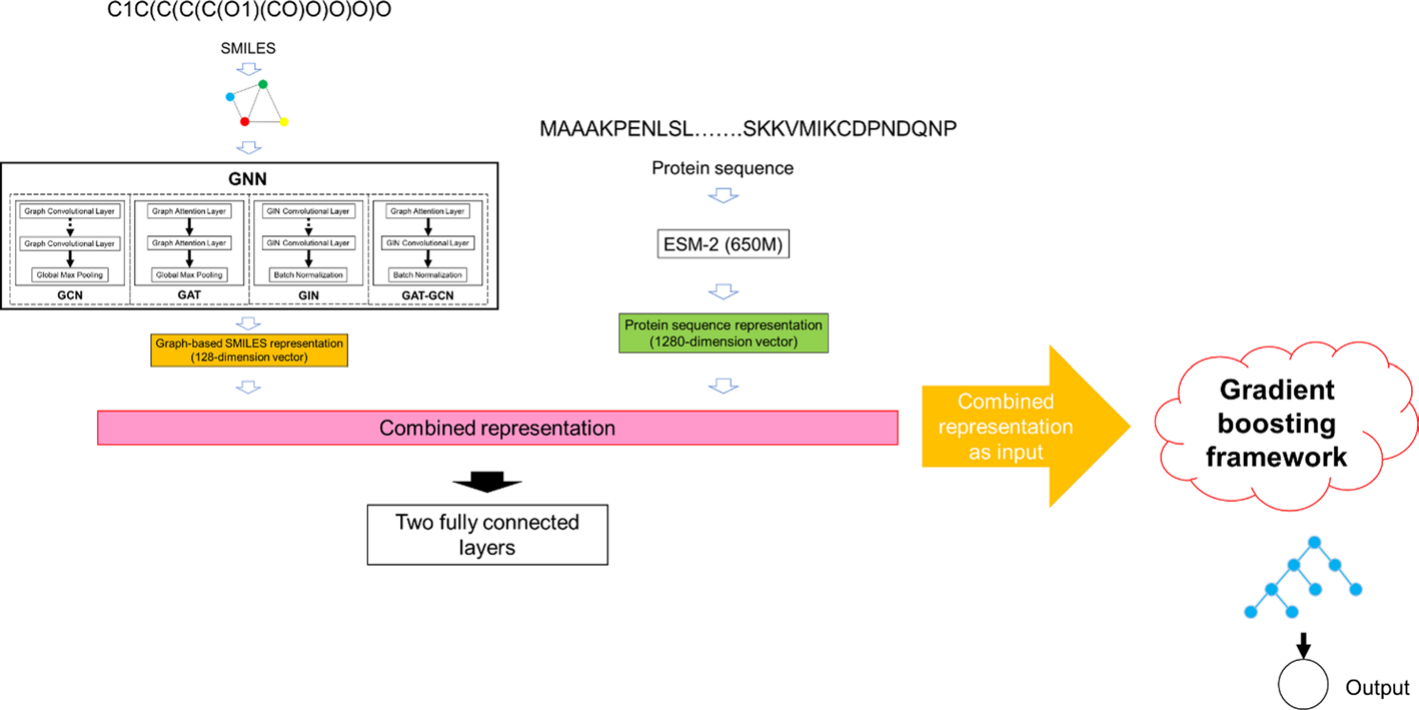
The rough architecture of the Grapheme model

The GraphKM is a machine and deep learning approach (Fig. 1). The training process of the GraphKM model was divided into two stages. In the first stage, the model was training with GNN and two FCNN layers. We used 4 different GNN (GIN, GCN, GAT and GAT-GCN). In the second stage, we fitted the gradient boosting framework for the output.

We used the molecular graph of substrate as the input of the GNN (i.e., GCN, GAT, GIN, or a combined GAT-GCN architecture). The GNN generated a 128-dimensional vector of substrate representation. Note that the four types of GNN are the common layers defined in Paddle Graph Learning (PGL) package (https://github.com/PaddlePaddle/PGL), which is an efficient and flexible graph learning framework implemented by the PaddlePaddle.

In the first stage, the output of the GNN was combined with protein representation into a 1,408-dimensional vector. The vector was used as the input of the two FCNN layers. The model was trained to the best model in 200 epochs with learning rate controlled by the Cosine Annealing Decay function, batch size 128, and 4 workers for PGL dataloader.

In the second stage, we used the gradient boosting framework fitting the substrate and enzyme information (a 1,408-dimensional vector) to predict ${K}_{M}$ values. Hyperopt tool was used for hyperparameter optimizations of the gradient boosting framework.

### Performance evaluation parameters for the GraphKM model

To make the comparison of training results, we used the performance metrics: coefficient of determination (${R}^{2}$, the larger the better) and Root Mean Square Error (r.m.s.e., the smaller the better).

The ${R}^{2}$ (Eq. 1) was calculated by scikit-learn [30] package.

1 $$\begin{array}{*{20}c} {R^{2} = 1 - \frac{{\mathop \sum \nolimits_{i = 1}^{n} \left( {y_{ie} - y_{ip} } \right)^{2} }}{{\mathop \sum \nolimits_{i = 1}^{n} \left( {y_{ie} - \overline{y}_{e} } \right)^{2} }}} \\ \end{array}$$

where ${y}_{ip}$ is the predicted ${K}_{M}$ value, ${y}_{ie}$ is the experimental ${K}_{M}$ value, ${\overline{y} }_{e}$ is the average of the experimental ${K}_{M}$ values and n is the total number of items in the test dataset.

The best model was chosen according to the minimal r.m.s.e. The r.m.s.e. (Eq. 2) was calculated by NumPy package.

2 $$\begin{array}{*{20}c} {r.m.s.e = \sqrt {\frac{1}{n}\mathop \sum \limits_{i = 1}^{n} \left( {y_{ip} - y_{ie} } \right)^{2} } } \\ \end{array}$$

We used the linear correlation coefficient (Pearson’s r) (Eq. 3) to evaluate the prediction performance of the models.

3 $$\begin{array}{*{20}c} {Pearson^{\prime}s\;r = \frac{1}{n - 1}\mathop \sum \limits_{i = 1}^{n} \left( {y_{ip} - \overline{y}_{p} } \right)\left( {y_{ie} - \overline{y}_{e} } \right)} \\ \end{array}$$

where ${\overline{y} }_{p}$ is the average of the predicted ${K}_{M}$ values.

### Independent dataset collection

We manually collected an independent dataset, namely HXKm, from literatures (Additional file 2: Table S1). As some entries with the same amino acid sequence and substrate have multiple ${K}_{M}$ values, we reserved the entry with the maximum ${K}_{M}$ value. We checked the overlap of HXKm dataset with the training set of the cleaned dataset via counting duplicates (i.e., entries with identical substrate and amino acid sequence as another entry). And we removed the duplicates. The final dataset consists of 443 entries with EC 1, 2, 3, 4, 5, and 6 class. Because the catalysis process of the Translocases (EC 7) is present for the movement of ions or molecules across membranes or their separation within membranes, the EC 7 class is not discussed in this study.

## Results

### Cleaned dataset

The cleaned dataset contains 19,754 unique entries, 11,314 entries with wildtype enzymes and 8,440 entries with mutant enzymes. Each entry contains substrate name, substrate SMILES code, EC number, protein sequence, organism name and ${K}_{M}$ value. We split the cleaned dataset into training set (80%) and test set (20%). The training set consists of 9,051 entries with wildtype enzymes and 6,752 entries with mutant enzymes. The test set consists of 2,263 entries with wildtype enzymes and 1,688 entries with mutant enzymes.

### The training results of the GraphKM models

**Fig. 2:**
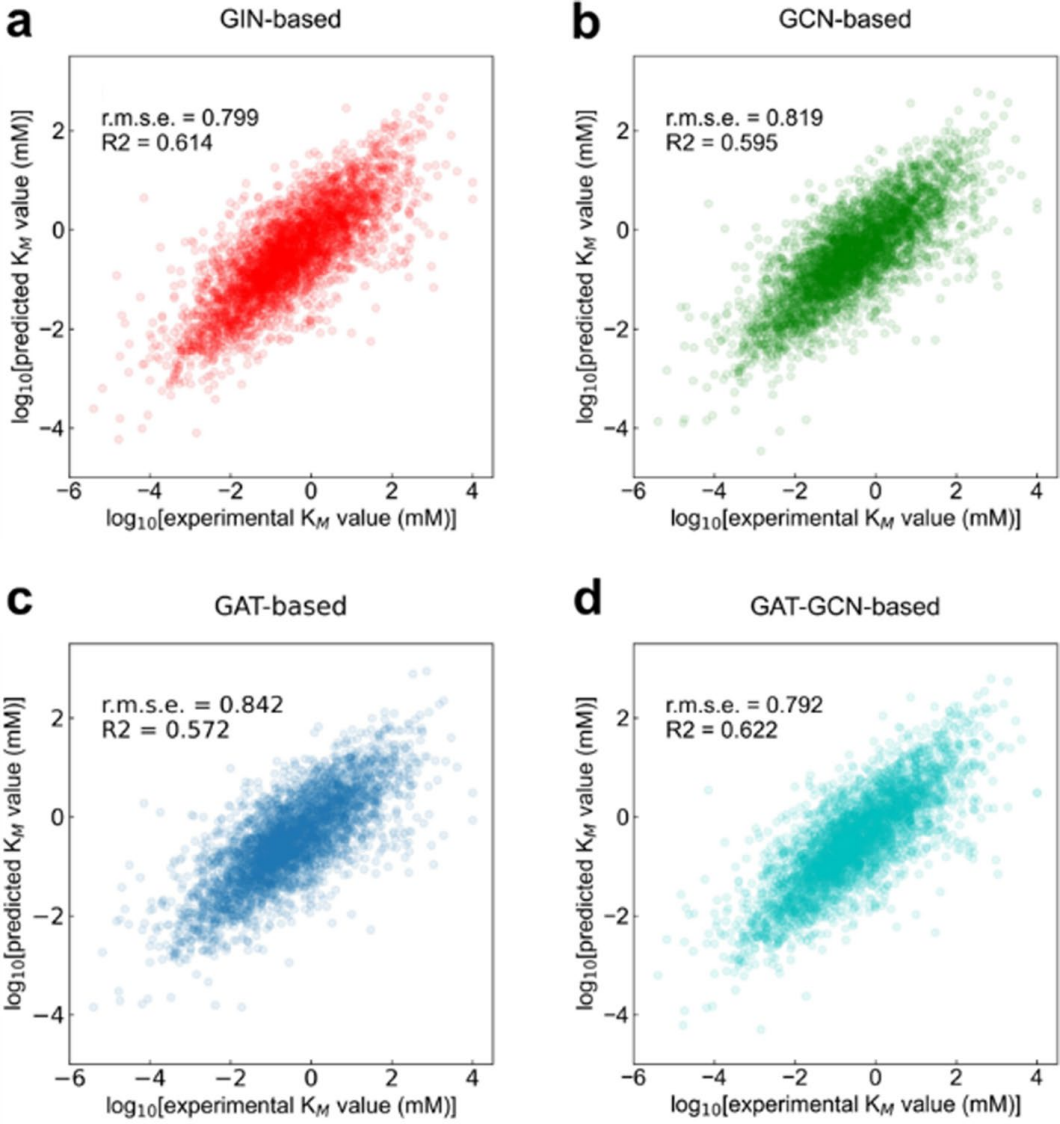
The performance of the GraphKM models

Protein sequences were represented as 1,280-dimensional vectors by ESM-2 tool, and substrates were represented as molecular graphs converted from SMILES. GNN is attractive to process substrate representation [7]. It is hypothesized that the fine-tuning of GNN will have an impact on the model results. We used different GNN (GIN, GAT, GCN, and GAT-GCN) present in PGL package (https://github.com/PaddlePaddle/PGL) to process the molecular graphs of substrates. The GAT-GCN-based model achieved a better performance (${R}^{2}$ = 0.622 and r.m.s.e. = 0.792) (Additional file 1: Fig. S2, Fig. 2).

### Prediction performance of the GraphKM models on data of different enzymes of the test set

#### Prediction performance of the GraphKM models on the data of wildtype and mutant enzymes

**Fig. 3:**
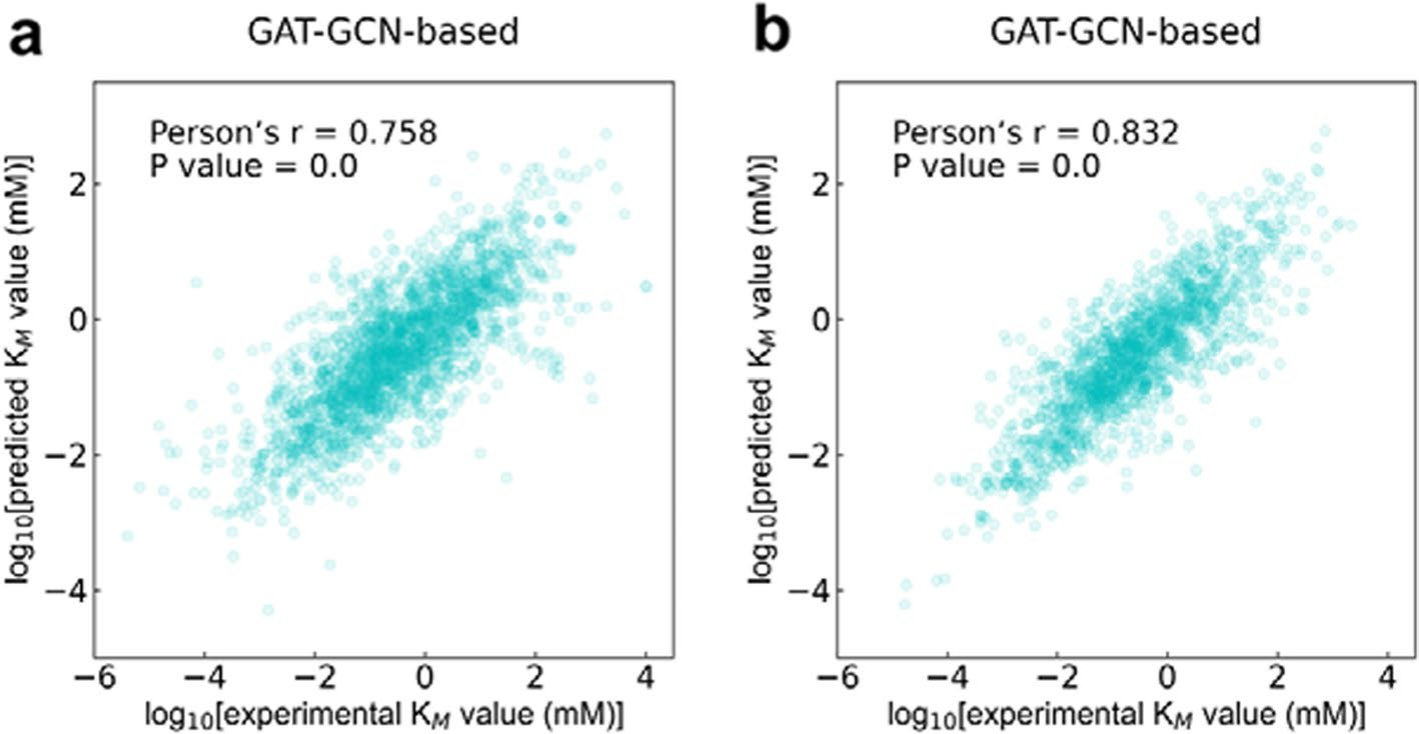
The correlation figures plotted between ${{\text{K}}}_{{\text{M}}}$ values predicted by the GAT-GCN-based model and the true values present in the test set (for the wildtype enzymes (**a**); for the mutant enzymes (**b**))

The test set of the cleaned dataset has data of wildtype and mutant enzymes. It is hypothesized that the performance of the models on the data of wildtype enzymes and the data of mutant enzymes is consistent with the results of the models on the whole test set (i.e. the GAT-GCN-based model still outperformed) (Fig. 2). We display the prediction performance of the models on the data of wildtype enzymes and the data of mutant enzymes (Additional file 1: Fig. S3, Fig. 3). As p values are all less than 0.05, the performance of GraphKM models on the data of wildtype and mutant enzymes are statistically significant. The GAT-GCN-based model performed better for both the data of wildtype enzymes (Fig. 3a Person’s r = 0.758, p = 0.0) and the data of mutant enzymes (Fig. 3b Person’s r = 0.832, p = 1.89 × 10^–76^). This result is consistent with the above hypothesis, and indicates that the data of wildtype enzymes and the data of mutant enzymes have limited effect on the performance of the models. As the data of wildtype enzymes is more than the data of mutant enzymes in both training set and test set (see Sect. "Cleaned dataset"), the models all outperformed on the data of mutant enzymes (Fig. 3 and Additional file 1: Fig. S3). This result indicates that the models are more suitable predicting for mutant enzymes.

#### Prediction performance of the GraphKM models on the data with different EC class

**Fig. 4:**
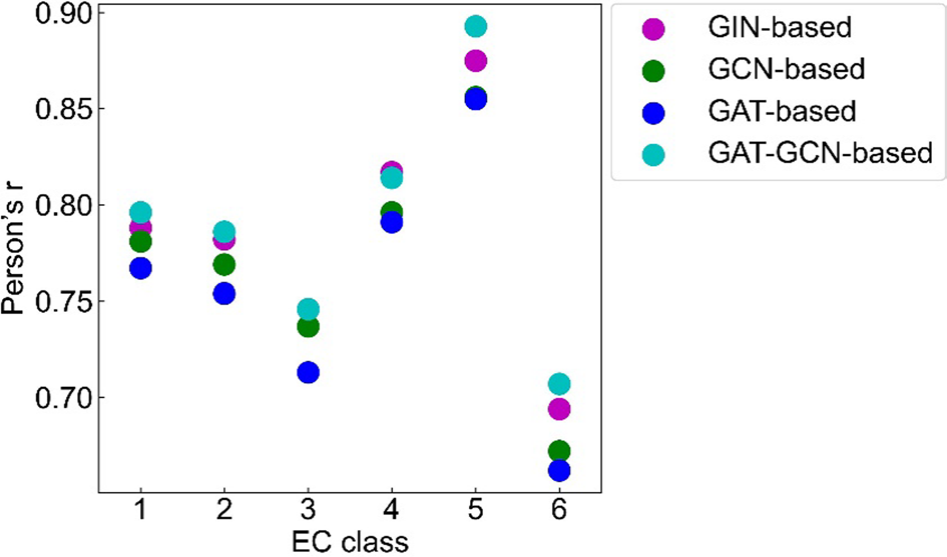
The prediction performance of the GIN-based, GCN-based, GAT-based and the GAT-GCN-based models on the data (with different EC class) of the test set

In the training set and test set, the order of the enzymes’ ratio is the same: EC 1 > EC 2 > EC 3 > EC 4 > EC 6 > EC 5 (Additional file 1: Fig. S4). It is hypothesized that the performance of the models on the data of enzymes with any EC class is consistent with the results of the models on the whole test set (i.e. the GAT-GCN-based model still outperformed) (Fig. 2). We display the prediction performance of the models on the data of enzymes with different EC class (Fig. 4, Additional file 1: Table S2). The GAT-GCN-based model performed better on the data with EC 1, 2, 3, 5, and 6 class. The GIN-based model performed better on the data with EC 4 class. This result is inconsistent with the above hypothesis, and indicates that the data of enzymes with the specific EC class have significant effect on the performance of the models (Fig. 2). The models all performed similarly with Pearson’s r values ordering from highest to lowest: EC 5 > EC 4 > EC 1 > EC 2 > EC 3 > EC 6 (Fig. 4). The amount of data with enzymes with the specific EC class is not correlate with the prediction performance of the models.

### The prediction performance of GraphKM and KM_prediction on the HXKM dataset

**Fig. 5:**
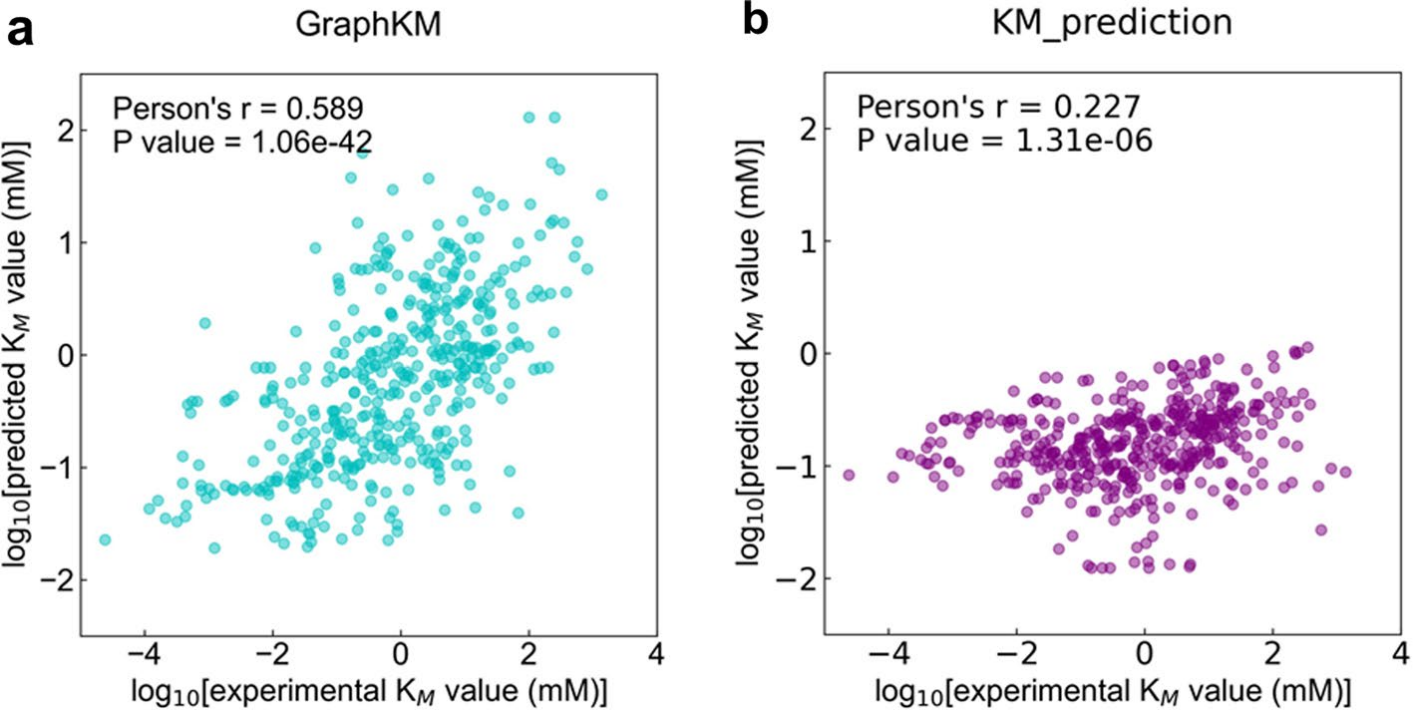
The correlations between ${{\text{K}}}_{{\text{M}}}$ values predicted by the models and true values present in the HXKM dataset

The training set of the cleaned dataset contains different enzymes. It is assumed that GraphKM can predict the ${K}_{M}$ values of different enzymes with their reported substrates. It is desirable to test GraphKM prediction performance on the data from an independent dataset. We collected the HXKM dataset (see Sect. "Independent dataset collection"), keeping only entries that were not already included in the training set. Each entry contains substrate name, substrate SMILES code, EC number, protein sequence, organism name, ${K}_{M}$ value, and PubMedID. GraphKM achieves a performance (Fig. 5a Person’s r = 0.589, p = 1.06 × 10^–42^) on the dataset. As p value is less than 0.05, the performance of GraphKM on the dataset is statistically significant. The entries of HXKM dataset were also not already included in the training set of KM_prediction. KM_prediction achieves a performance (Fig. 5b Person’s r = 0.227, p = 1.31 × 10^–6^) on the dataset. The performance of KM_prediction on the dataset is statistically significant.

### The prediction performance of GraphKM and MLAGO on the subset of HXKm dataset

**Fig. 6:**
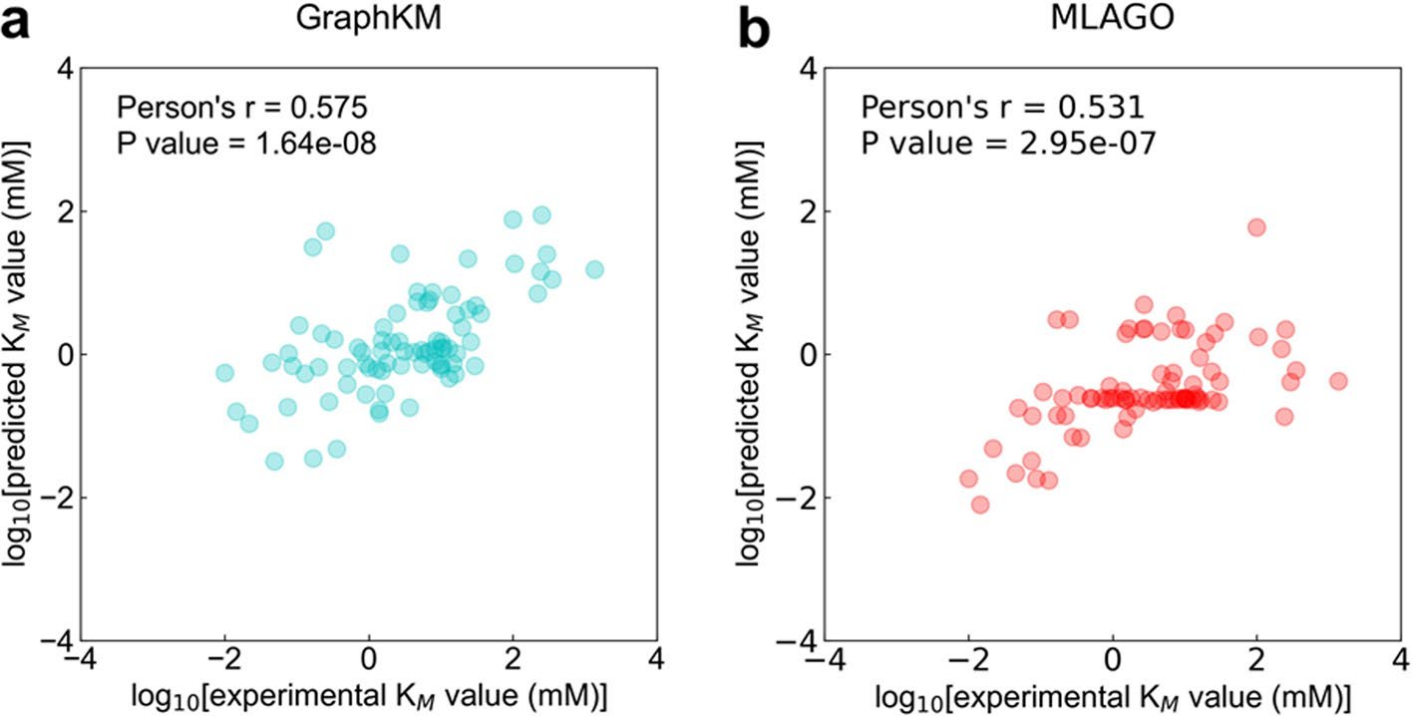
The correlations between ${{\text{K}}}_{{\text{M}}}$ values predicted by the models and true values present in the GMKM dataset

The inputs of MLAGO are one-hot encodings of EC number, Kegg Compound ID, and Organism ID, while the encoding rules were defined by the authors [10]. Some substrates and organism names of the HXKm dataset were not included in the encoding rules. We collected a new dataset from HXKm dataset, namely GMKM, containing 82 entries to evaluate the prediction performance of MLAGO and GraphKM (Additional file 3: Table S3). One entry contains EC number, amino acid sequence, substrate SMILES, Kegg Compound ID, organism ID etc. The entries of GMKM dataset were not already included in the training set of GraphKM and MLAGO. We took the one-hot encoding approach employed by Maeda et al. [10] to encode EC number, Kegg Compound ID, and Organism ID of the dataset (Additional files 4, 5, 6: Table S4–S6). On the dataset, GraphKM shows a performance (Fig. 6a Person’s r = 0.575, p = 1.64 × 10^–8^). MLAGO shows a performance (Fig. 6b Person’s r = 0.531, p = 2.95 × 10^–7^). The performance of GraphKM and MLAGO is statistically significant.

## Discussion

The parameters of enzyme catalytic properties, like ${K}_{M}$, are mainly collected in the BRENDA [25] and SABIO-RK [26] databases. In the databases, an enzyme for a specific substrate has multiple ${K}_{M}$ values, which are produced under different experimental procedures, such as temperature, pH value etc. Without considering the experimental procedures, only one ${K}_{M}$ value of the enzyme is used for model training. Faced with this situation, Kroll et al. took the geometric mean of the multiple values as ${K}_{M}$ label [7]. This way seems to take into account the ${K}_{M}$ variation, but the geometric mean is not the true value with the specific conditions. We chose the maximum ${K}_{M}$ value of the enzyme. We removed some enzymes of the databases that have amino acid sequences length more than 1000. These enzymes may have redundant structural domains and catalytic functions. These domains and functions may not be related to the catalytic function of the enzyme for a specific substrate and would interfere with our model training. There is no independent dataset of wildtype and mutant enzymes to test ${K}_{M}$ prediction models. We collected an independent dataset (HXKm) from literatures.

GNN may be more attractive than other substrate representation methods (e.g. ECFP, RDKit fingerprint, MACCS keys) [7]. We used different GNN (GIN, GAT, GCN, and GAT-GCN) present in PGL package (https://github.com/PaddlePaddle/PGL) to process substrate representation, which shows the prediction performance difference on the test set (Fig. 2). The reason for this difference may be that different GNN has its own characteristics when processing graph representations. The characteristics are better reflected in the prediction performance for different enzymes [29] (Fig. 3 and Additional file 1: Figs. S3 and S4).

The different prediction results (Fig. 3 and Additional file 1: Fig. S3 and S4) for different enzymes is likely due to the insufficient data amount of the cleaned dataset used for model training. It is believed that data amount of training set is positively correlated with prediction performance of model on test set [7]. The prediction performance of the models on the data with EC 1, 2, 3, and 6 class is consistent with this case. The prediction performance on the data with EC 3, 4, and 5 class (Fig. 4) contradicts this case. One reason for this result might be the insufficient data amount with the specific EC class of the cleaned dataset. The prediction performance on the data of wildtype and mutant enzymes (Fig. 3 and Additional file 1: Fig. S3) also contradicts this case. One reason for this result might be that the representation changes of mutant enzymes are more easily learned by the models.

KM_prediction used Kegg Compound ID to acquire the MDL Molfile of substrate from KEGG [31]. The MDL Molfile was the input of KM_prediction [7]. MLAGO took the one-hot encodings of EC number, Kegg Compound ID, and Organism ID as input [10]. The encoding rules were defined by the authors [10]. The way of encoding is not applicable for the EC number, Kegg Compound ID, or Organism ID beyond its training dataset. The usage of MLAGO is limited by its encoding rules [10]. GraphKM found a solution requiring only the substrate SMILES and amino acids sequences as input. On the independent HXKM dataset, GraphKM shows better prediction performance (Fig. 5a Person’s r = 0.589). GraphKM also shows a better prediction performance (Fig. 6a Person’s r = 0.575) on the independent GMKM dataset.

In conclusion, we used the PaddlePaddle to implement the deep learning model GraphKM to predict ${K}_{M}$ values of wildtype and mutant enzymes against their substrates, requiring only the substrate SMILES information and protein sequences of the enzymes as input.

### Supplementary Information

**Additional file 1.** Data cleaning process, the r.m.s.e. trend of model in training process, the correlation figures plotted KM values predicted by models and the true values present in the data of wildtype and mutant enzymes of test set, distribution of data of different enzymes training set and test set, and details of the prediction performance of models on the data of enzymes with different EC classification in test set.

**Additional file 2.** The independent dataset, HXKm.

**Additional file 3.** The subset of HXKm dataset, GMKM.

**Additional file 4.** The one-hot encodings of EC number.

**Additional file 5.** The one-hot encodings of Kegg compound ID.

**Additional file 6.** The one-hot encodings of Organism ID.

## Data Availability

The collected data and the Python codes used to generate all training results are publicly available only at https://github.com/realHXiao/GraphKM. The training and prediction results are available in the Figshare database with 10.6084/m9.figshare.25335049.
