# Supplementary material for: GraphKM: machine and deep learning for KM prediction of wildtype and mutant enzymes
Source: BMC Bioinformatics. 2024 Mar 28;25:135. doi: 10.1186/s12859-024-05746-1 (PMC10979596; doi:10.1186/s12859-024-05746-1)
Supplement: Supplementary file 1 — Additional file 1. Data cleaning process, the r.m.s.e. trend of model in training process, the correlation figures plotted KM values predicted by models and the true values present in the data of wildtype and mutant enzymes of test set, distribution of data of different enzymes training set and test set, and details of the prediction performance of models on the data of enzymes with different EC classification in test set. [file 12859_2024_5746_MOESM1_ESM.docx]

1. Data cleaning process for the GraphKM model input.


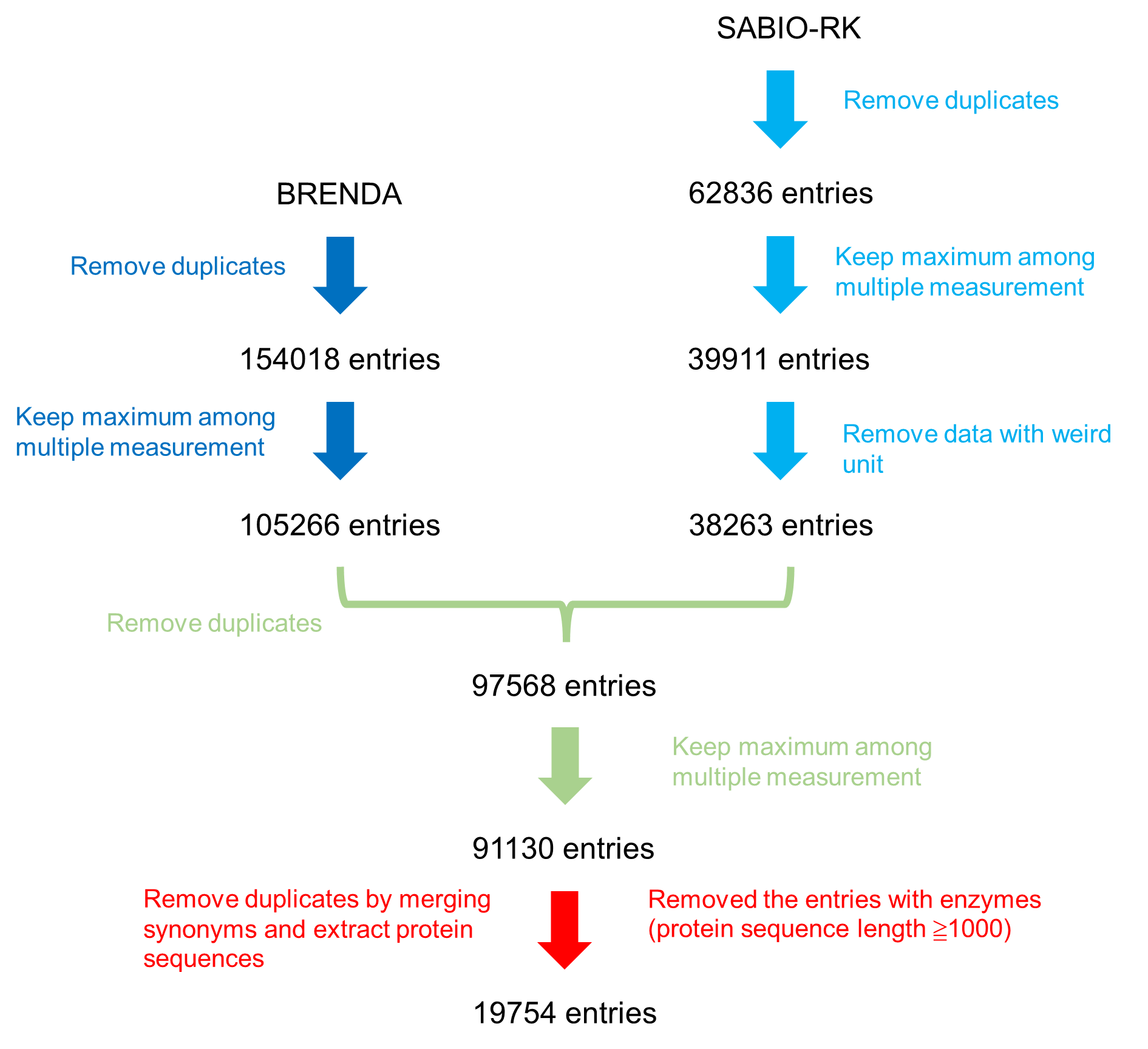


1. The trend of r.m.s.e of the GraphKM models in training process.


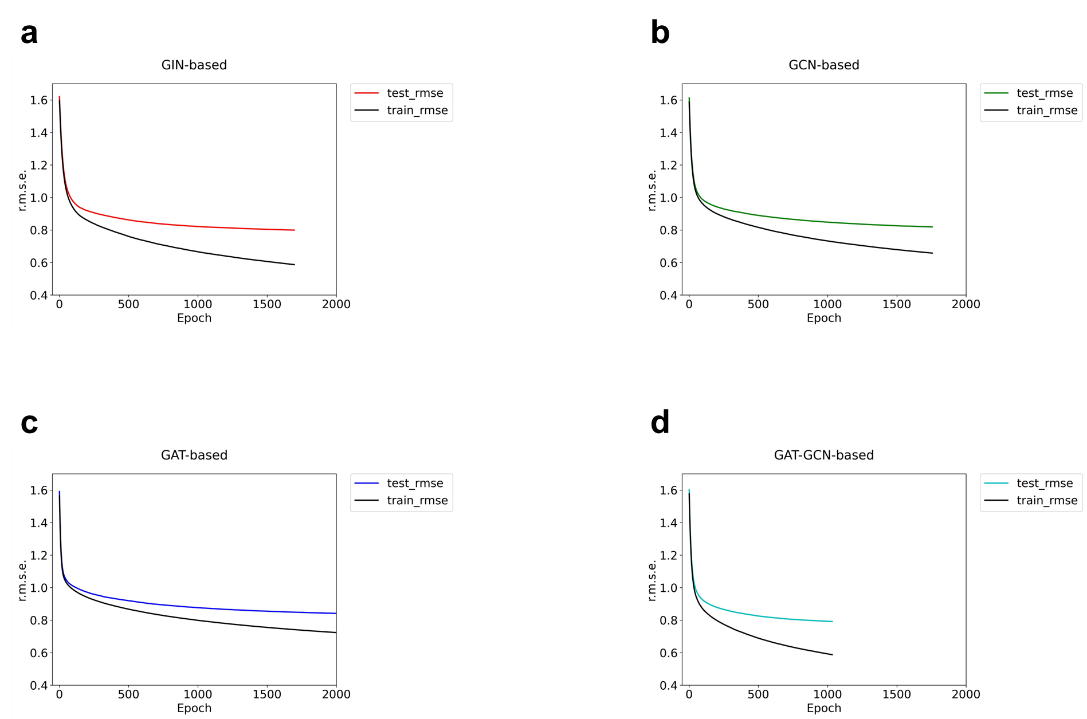


1. The correlation figures plotted between $K_{M}$ values predicted by the GraphKM models and the true values present in the cleaned dataset (for the data of wildtype enzymes in the test set (**a, b, c**); for the data of mutant enzymes in the test set (**d, e, f**)).


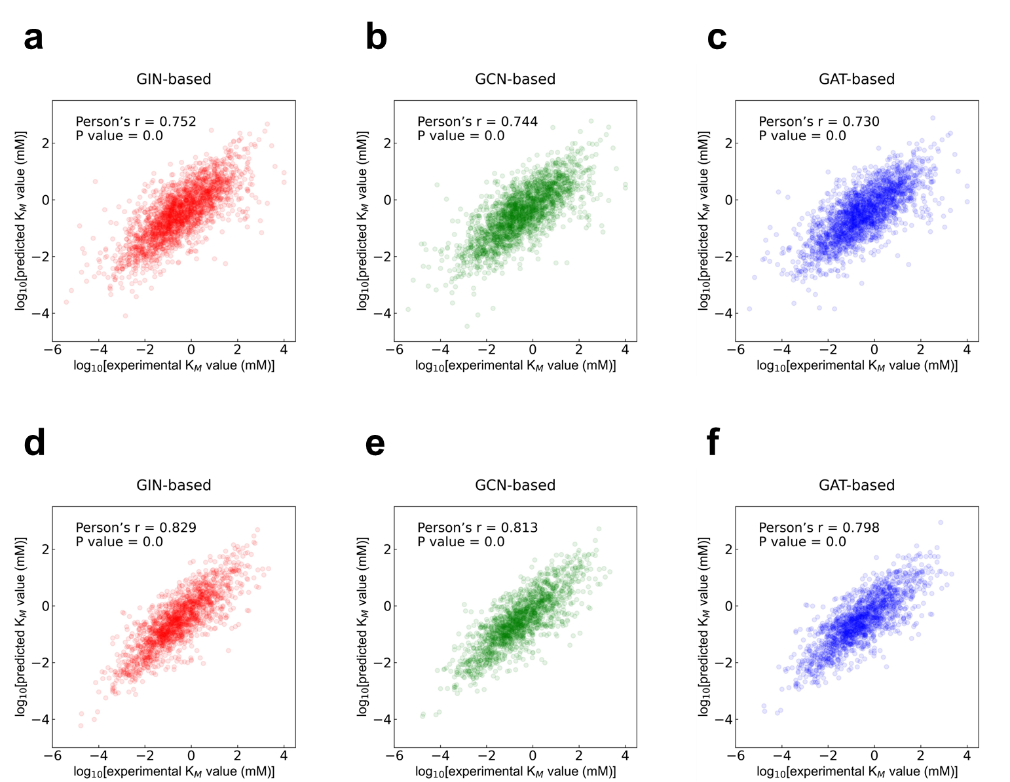


1. The distribution of data with the six EC class and the distribution of wildtype and mutant enzymes in the training set (**a**) and test set (**b**) of the cleaned dataset.


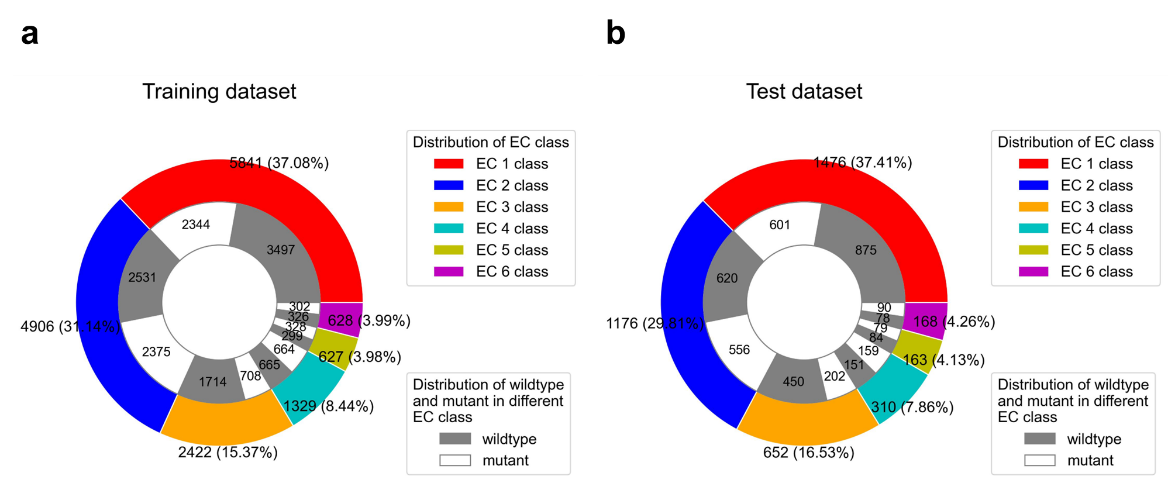


1. The prediction performance of the GraphKM models on the data with the different EC class in the test set of the cleaned dataset.

| EC class | Prediction performance of GIN-variant | |
| --- | --- | --- |
|  | Person’s r | P value |
| 1 | 0.788 | 2.67×10^-313^ |
| 2 | 0.782 | 4.22×10^-243^ |
| 3 | 0.746 | 5.26×10^-117^ |
| 4 | 0.817 | 1.21×10^-75^ |
| 5 | 0.875 | 1.82×10^-52^ |
| 6 | 0.694 | 2.00×10^-25^ |
| EC class | Prediction performance of GCN-variant | |
|  | Person’s r | P value |
| 1 | 0.781 | 6.61×10^-304^ |
| 2 | 0.769 | 2.98×10^-230^ |
| 3 | 0.737 | 1.29×10^-112^ |
| 4 | 0.796 | 4.66×10^-69^ |
| 5 | 0.856 | 6.66×10^-48^ |
| 6 | 0.672 | 2.14×10^-23^ |
| EC class | Prediction performance of GAT-variant | |
|  | Person’s r | P value |
| 1 | 0.767 | 8.20×10^-286^ |
| 2 | 0.754 | 4.38×10^-216^ |
| 3 | 0.713 | 3.60×10^-102^ |
| 4 | 0.791 | 9.96×10^-68^ |
| 5 | 0.855 | 8.64×10^-48^ |
| 6 | 0.662 | 1.62×10^-22^ |
| EC class | Prediction performance of GAT-GCN-variant | |
|  | Person’s r | P value |
| 1 | 0.796 | 3.00×10^-323^ |
| 2 | 0.786 | 5.30×10^-247^ |
| 3 | 0.746 | 1.07×10^-116^ |
| 4 | 0.814 | 1.44×10^-74^ |
| 5 | 0.893 | 1.14×10^-57^ |
| 6 | 0.707 | 1.00×10^-26^ |
